# Using Generalized Procrustes Analysis (GPA) for normalization of cDNA microarray data

**DOI:** 10.1186/1471-2105-9-25

**Published:** 2008-01-16

**Authors:** Huiling Xiong, Dapeng Zhang, Christopher J Martyniuk, Vance L Trudeau, Xuhua Xia

**Affiliations:** 1 Centre for Advanced Research in Environmental Genomics (CAREG), Department of Biology, University of Ottawa, Ottawa, Ontario, K1N 6N5, Canada

## Abstract

**Background:**

Normalization is essential in dual-labelled microarray data analysis to remove non-biological variations and systematic biases. Many normalization methods have been used to remove such biases within slides (Global, Lowess) and across slides (Scale, Quantile and VSN). However, all these popular approaches have critical assumptions about data distribution, which is often not valid in practice.

**Results:**

In this study, we propose a novel assumption-free normalization method based on the Generalized Procrustes Analysis (GPA) algorithm. Using experimental and simulated normal microarray data and boutique array data, we systemically evaluate the ability of the GPA method in normalization compared with six other popular normalization methods including Global, Lowess, Scale, Quantile, VSN, and one boutique array-specific housekeeping gene method. The assessment of these methods is based on three different empirical criteria: across-slide variability, the Kolmogorov-Smirnov (K-S) statistic and the mean square error (MSE). Compared with other methods, the GPA method performs effectively and consistently better in reducing across-slide variability and removing systematic bias.

**Conclusion:**

The GPA method is an effective normalization approach for microarray data analysis. In particular, it is free from the statistical and biological assumptions inherent in other normalization methods that are often difficult to validate. Therefore, the GPA method has a major advantage in that it can be applied to diverse types of array sets, especially to the boutique array where the majority of genes may be differentially expressed.

## Background

The cDNA microarray is a widely used high-throughput technique for gene expression profiling, especially for organisms whose genome sequences are unavailable. However, in microarray experiments, there exist many non-random variations and systematic biases, which can confound the extraction of the true fluorescence intensity signals, and thus compromise downstream data analysis and interpretation of the experimental data. Therefore, proper data normalization is required to remove these biases before accurate identification of differential gene expression [1-4].

The main objective of normalization is to ensure that measured intensities within and across slides are comparable. Based on different biological or statistical assumptions about data distribution or experimental design, various normalization methods have been proposed. The housekeeping gene method [5] is an early normalization method, which assumes that the expression levels of housekeeping genes remain constant even when the expression of many other genes is substantially changed. However, many so-called housekeeping genes have been reported to exhibit considerable variability under different experimental conditions and different tissues [6], making them unsuitable and unrepresentative of the whole expression intensity range. The Global normalization approach [5] assumes that the center (mean or median) of the distribution of log ratio M values in each slide is zero. However, the Global normalization method does not consider intensity-dependent and spatially-dependent effects, which are usually major biases among the slides. In order to remove such biases, Yang et al. [4] proposed one local regression smoothing procedure (Lowess) that is applied to each slide separately to normalize the log ratio intensities. Lowess normalization has been one of the most popular methods but it has two important assumptions. Lowess assumes that most genes on the array are not differentially expressed across the experiments and also that the numbers of up- and down-regulated genes at each intensity level are roughly equal in each slide. Other methods including the semiparametric [2], neural network [7], and common array dye-swap methods [8] have been proposed to remove intensity-dependent biases.

These various methods can effectively remove the intensity-dependent or spatially-dependent biases within each slide. However, they do not account for the intensity-dependent differences across multiple slides, which can introduce undue weighting of some slides to an average of log-ratios across slides in the subsequent data analysis [4]. Scale normalization [4] is one popular approach for such across-slide normalization [3,9], in which log ratio intensities are assumed to follow a normal distribution with expectation zero and homogeneity of variance across replicated arrays. Other effective across-slide normalization methods include Quantile [10] and Variance stabilization normalization (VSN) [11]. Quantile normalization was initially developed for the Affymetrix single channel chip [10], and then extended for two colour cDNA microarrays in the Limma package of the Bioconductor project [12]. It relies on the assumption that the probe intensities for each array in a set of replicated arrays are approximately equally distributed. The goal therefore is to adjust for the difference in distribution among multiple slides, and data points are shifted such that the sample densities of slides are identical. In contrast, the VSN method assumes that most of the genes on the arrays are not differentially expressed in a given experiment and utilizes the arcsine rather than log transformation to stabilize the variance so as to remove the dependence of the variance on the total intensity. This gives genes with higher intensities an equal chance of being ranked high as genes with lower intensity. VSN has been used for both the Affymetrix [13] and cDNA microarray platforms [14].

While many different normalization methods are available and diverse strategies are implicated, most of them require certain critical biological or statistical assumptions about data distribution. For example, one usual assumption underlying the Global, Scale, Lowess and VSN methods is that the array contains many non-differentially expressed genes. The assumption on data distribution inherent in these methods may not be valid in practice. For example, in custom-made boutique arrays most of genes are expected to be differentially expressed [15,16]. Most of the above normalization methods are inappropriate. Although some novel methods have been proposed for such boutique arrays, including the housekeeping gene [15], Zipf's law [17] and the mixture model based methods [18], they still have their own assumptions. The commonly used housekeeping gene method assumes that a set of prior housekeeping genes exists in the microarray in similar expression patterns, and could be utilized for Lowess normalization. However, the hybridization signals from these proposed housekeeping genes may not span the entire fluorescence range produced by boutique arrays. Zipf's law method [17] assumes that the microarray data set exhibits an observed power-law distribution with an exponent close to -1, whereas the mixture model based method [18] assumes that the log ratio of intensity in each channel has a Gamma distribution. Although many available normalization methods are effective for removing some types of biases among arrays, their own assumptions about data distribution may limit their application, or even introduce new biases. There is a clear need for developing versatile and robust methods for normalization of microarray data.

Here we present evidence to show that the Generalized Procrustes Analysis [19-21], or GPA, is a powerful statistical method for normalization of microarray data. The GPA method differs from other methodologies in that it has no assumptions about data distributions, which delineates its main advantage over the other methods. Procrustes analysis is one of the least-squares methods for translation, rotation, scaling and aligning matrices of corresponding coordinates to maximize their agreement [19-21]. The transformation parameters are computed in a direct and efficient manner based on a selected set of corresponding point coordinates. Microarray data are matrices of colour intensities. Replicated slides are matrices of similar configurations and are therefore amenable to Procrustes analysis. On the basis of publicly available and simulated data, our GPA-based normalization strategy is systemically compared with six other popular normalization methods including two within-slide methods (Global and Lowess), three across-slide methods (Scale, Quantile and VSN), and one boutique array specific housekeeping gene method. The assessment of these methods is based on three empirical criteria: variability among replicated slides [3,22], the Kolmogorov-Smirnov statistic [17,23] and the mean square error [10].

## Results

### Generalized Procrustes Analysis (GPA) in normalization of microarray data

**Figure 1:**
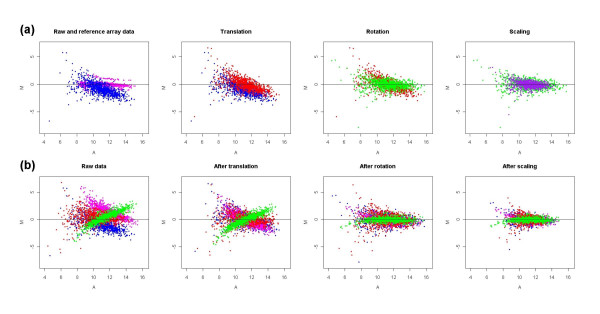
**A geometric transformation of microarray M-A plots in GPA normalization**. (a) shows how the M-A plot for one slide transformed during each GPA transformation procedure. The blue points represent raw data; pink points represent reference slide; red, green and purple points represent data points after translation, rotation and scaling, respectively; (b) shows how the M-A plots for four slides represented by four colours (blue, red, pink, green) transformed after each GPA transformation procedure. The SIMAGE method was used to simulate the microarray data set used here, which includes 50 slides with 10% differentially expressed genes and ratio of up-regulated to down-regulated genes is 1:1.

GPA is a standard multivariate statistical method widely applied in shape analysis to find the optimal superimposition of two or multiple configurations [19-21]. The algorithm involves three transformations: translation, rotation and scaling. Translation is a movement in which the centroid of each configuration is shifted to the common origin by subtracting centroid coordinate. Rotation is a fixed displacement of all points by a constant angle, keeping the distance of each point from the centroid unchanged. Scaling is a stretching or shrinking of all points by a constant amount in a straight line from the point to the centroid of the configuration. The optimal transformation is defined as one with the smallest sum of the squared distances among corresponding points in the configurations. In our study GPA method is used to minimize the deviation of signal intensities among microarray slides. A detailed geometric transformation of microarray M-A plots in GPA normalization is illustrated in Figure 1 with one set of simulated microarray data. Figure 1(a) shows the transformations for one slide and Figure 1(b) shows the superimposition among multiple slides (4 here) after each GPA transformation procedure. Two characters of GPA normalization are that it does not change the relative position of points (genes) within each M-A plot and the transformations (translation, rotation and scaling) are based on a global optimization instead of local optimization.

### Strategy for comparison of normalization methods

Based on the different data sets, we compared our GPA-based method with six other normalization methods, including Global [5], Lowess [4], Scale [4], Quantile [10], VSN [11], and the housekeeping gene method for boutique array [15]. The comparison includes three levels. We firstly evaluated the performance of these different normalization methods in removing biases individually, no matter what strategies are behind them. Thereafter, we systematically compared several pairs of within-slide and across-slide normalization methods to (1) evaluate the potential of combinations of different methods in normalization procedures, and (2) assess the ability of decreasing across-slide bias of the GPA method compared to other across-slide normalization methods based on the same within-slide normalization background. This bears significance in practice since it is often necessary to combine different normalization strategies to decrease variation and potential bias within each slide and across slides. Thirdly, for the boutique data in which common normalization methods are not suitable, we evaluated the GPA method with the housekeeping gene normalization method without other methods involved.

We first evaluated the performance of these normalization methods on two real microarray data sets through comparing data variability and similarity of data distribution among replicated slides. The rationale behind these two criteria is that an effective normalization method should result in lower replicate variability, and more similar (ideally identical) data distributions for replicated slides. Then we applied two simulation methods to simulate several types of microarray data. Mean square error (MSE) was used to evaluate different normalization methods through calculating the true difference between simulated data and normalized data. For the boutique array type, both real data and simulated data were utilized and subjected to these three empirical criteria. In addition, the differential effects of these normalization methods on M-A plots for both real data and simulated data are showed in Additional files 1, 2.

### Comparison based on the criterion of replicate variability

The criterion of replicate variability is based on the rationale that the expression level of a gene should ideally remain the same across multiple replicated slides. For *N *replicated slides, the variability of *N *values for each gene can therefore be used to compare normalization methods [3,22]. The standard deviation for gene *g *(*σ*_g_) can be estimated as

(3) $${\overset{⌢}{\sigma }}_{g}=\sqrt{\frac{1}{N-1}\sum_{i=1}^{N}(M_{gi}^{*}-{\bar{M}}_{g\cdot }^{*}{)}^{2}}$$

where *N *is the number of replicates in the data set, $M_{gi}^{*}$ is the normalized *log*_2_(*R*/*G*) value for gene *g *in slide *i*, while ${\bar{M}}_{g.}$ is the average log ratio intensity over the slides for gene *g*. A smaller ${\hat{\sigma }}_{g}$ is indicative of a more effective normalization procedure. The mean of such ${\hat{\sigma }}_{g}$ estimates over all genes is a global measure of the performance of the normalization methods, with smaller mean ${\hat{\sigma }}_{g}$ indicative of better performance of the normalization methods.

**Figure 2:**
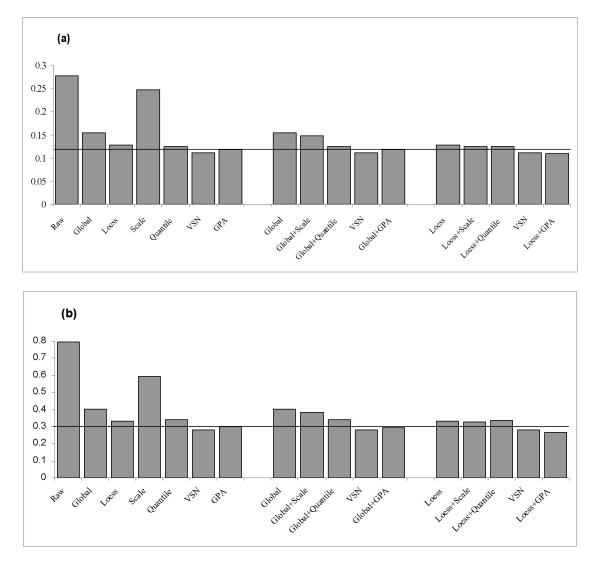
**Mean of replicate variability **${\hat{\sigma }}_{g}$**for the (a) swirl zebrafish data set and (b) HCT116 data set**. Larger value indicates a higher variability across slides. The reference line indicates the variability value for the GPA method.

Figure 2 shows bar plots of the variance estimates for the (a) swirl zebrafish and (b) HCT116 cancer data sets. Each bar represents the mean value of replicate variability ${\hat{\sigma }}_{g}$ for all genes. For both data sets, all normalization methods decrease variability of the raw data. However, the GPA method alone yields lower variability than the Lowess, Quantile, Global, and Scale methods do. A Wilcoxon test indicates that the differences are significant (p < 0.01). Here the VSN method performs better than the GPA method, which is expected because VSN method specifically aims to stabilize the variance across the replicated arrays.

When we compare the across-slide normalization methods based on the same within-slide normalization background (after Global or Lowess normalization), we can see (1) different combinations of within- and across-slide normalization methods can further reduce the variability values (Figure 2), and (2) the GPA method can result in a greater decrease than Scale and Quantile do (p < 0.01). It is particularly evident in the combination of Lowess and GPA methods compared with other dual normalizations including Global-Scale, Global-Quantile, as well as Lowess-Scale, Lowess-Quantile pairs. It is also somewhat better than that of the VSN method (0.1102 in swirl data and 0.2793 in HCT116 cancer data) (p < 0.01). In conclusion, our GPA method provides greater reduction of replicate error individually and in combination with other methods such as Lowess.

### Comparison using the Kolmogorov-Smirnov (K-S) test

The Kolmogorov-Smirnov (K-S) test is a goodness-of-fit test of two continuous distributions. It is used as a criterion for assessing normalization methods and is based on the rationale that an effective normalization procedure should result in two similar (ideally identical) distributions with a small, ideally zero-valued, K-S statistic [17,23]. In contrast, two different distributions will generate a large K-S statistic.

**Figure 3:**
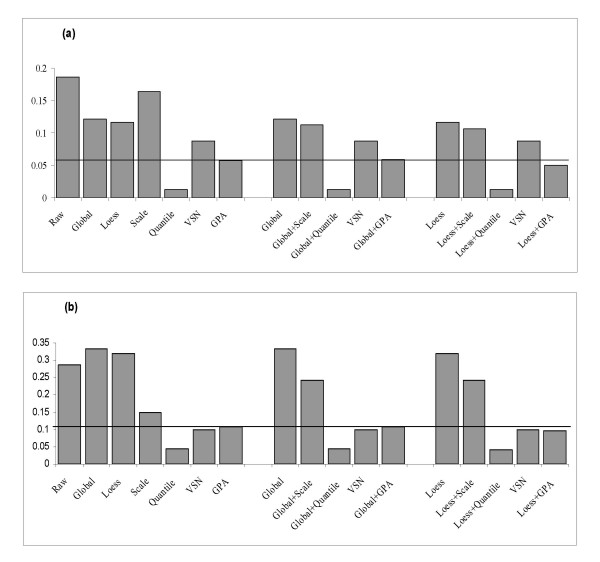
**Mean of K-S statistic between pairs of slides for the (a) swirl zebrafish data set and (b) HCT116 data set**. The reference line indicates the K-S value for the GPA method.

Figure 3(a) shows that for the swirl data set the K-S statistic for the GPA normalization method is much lower than that for the Global, Lowess, Scale and VSN methods alone. However, as expected, the K-S statistic is lowest with Quantile which forces the empirical distributions in different slides to be identical. This also reveals that the Quantile method is an aggressive normalization process as originally noted by [10]. Except for Quantile normalization, the different combinations of within- and across-slide normalization methods show that Scale, VSN, GPA can decrease the discrepancy of data distribution after within-slide normalization (Global or Lowess), but GPA effect is particularly evident (Figure 3(a)). The combination of Lowess and GPA methods produces a lower K-S value than other methods or method pairs. A similar result can be observed with the HCT116 cancer data set (Figure 3(b)) except that VSN is slightly better than GPA alone, but is somewhat outperformed by the Lowess-GPA pair (0.1003 vs. 0.097). For the HCT116 cancer data, although variability is decreased after Lowess or Global normalizations (Figure 2(b)), and an ideal M-A plot is produced after Lowess normalization, the discrepancies of data distribution among slides are increased. This is an example that the contributions of within-slide based normalization (Lowess) and across-slide based normalization differ in various data types. Overall, the GPA method results in a more similarly distributed data than the other four normalization methods.

### Comparison using the mean square error (MSE) criterion

We used a data simulation study to further test the GPA method. The advantage of using a simulated data set is that the true intensities are known, so we can assess the accuracy and precision of normalized data more systematically. Mean square error (MSE) is a widely used comparison criterion, which can calculate the true difference between simulated and normalized data [10,24]. Denote *M*_*ji *_as the true expression log-ratio for the *j*-th gene of *i*-th replicate, ${\hat{M}}_{ji}$ as the estimated value for *M*_*ji*_, and ${\bar{M}}_{j.}$ as the mean of ${\hat{M}}_{ji}$(*i *= 1,2, ...*N*, *j *= 1, 2, ..., the number of genes) in *N *replicates. MSE for the *j*-th gene is defined as

(4) $$\begin{array}{c} MSE_{j}=\frac{1}{N}\sum_{i=1}^{N}{\left(M_{ji}-{\hat{M}}_{ji}\right)}^{2}\\ =\frac{1}{N}\sum_{i=1}^{N}{\left({\hat{M}}_{ji}-{\bar{M}}_{j.}\right)}^{2}+\frac{1}{N}\sum_{i=1}^{N}{\left(M_{ji}-{\bar{M}}_{j.}\right)}^{2}\\ =Var\left({\hat{M}}_{ji}\right)+bias^{2}\left(M_{ji}\right) \end{array}$$

Note that MSE is decomposed into variance and squared bias. The variance component (*ν*) is an index of precision and the bias component (*β*) is an index of accuracy. Obtaining data with satisfactory precision and accuracy has been one of the biggest challenges in the application of microarrays. For this reason, MSE is an excellent criterion for evaluating alternative normalization methods [3,10], with smaller *ν *and *β *values indicating better normalization.

Two different methods to simulate microarray data were used: one is a parameterized random signal model from the study of [25], which is flexible and has been widely utilized; the other is recently published SIMAGE method [26], which simulates microarray data based on the estimated parameters from real microarray data. Based on the first method, two types of data are simulated, one without dye bias and one with the banana-shaped dye bias. Four different levels (3, 5, 10 and 30%) of differentially expressed genes are considered. The ratio of the up-regulated genes to the down-regulated genes is 1:1. The MSE values for the data set with 5% differential genes following different normalization methods are shown in Table 1. In both cases, the median of variance (*ν*) is several times lower with the GPA normalization individually than with the other normalization methods. The median of bias (*β*) with GPA normalization is also significantly lower than with the other methods (p < 0.01 by Wilcoxon test). This trend is observed for other data sets with 3, 10, 30% differentially expressed genes (Additional file 3). Based on the SIMAGE method, three sets of microarray data are simulated with 5, 10, 30 % differential expressed genes (up:down = 1:1). As shown in Table 2, our GPA method produced the lowest *ν *and *β *values compared with all other methods in three data sets (p < 0.01 by Wilcoxon test). These results indicate that the GPA method performs effectively and consistently better in reducing across-slide variability and removing systematic bias.

**Table 1:** Comparison among different normalization methods based on simulated normal microarray data with the Balagurunathan's method. The data sets are simulated without or with dye bias and include 1000 genes in 10 slides with 5% differentially expressed genes. The ratio of up-regulated to down-regulated genes is 1:1. The additional file 3 contains the full data sets.

**Method**	**Without dye bias**		**With dye bias**	
	**ν**^(1)^	**β**^(1)^	**ν**	**β**
Raw	0.1182	0.004627	0.1231	0.004586
Global	0.1179	0.004732	0.1223	0.00473
Lowess	0.1143	0.005368	0.1182	0.004976
Scale	0.1113	0.004666	0.1201	0.004515
Quantile	0.1149	0.005226	0.1212	0.004716
VSN	0.06294	0.002581	0.06314	0.002541
GPA	0.02122	0.00117	0.02069	0.00119
Global+Scale	0.1117	0.004579	0.1191	0.004595
Global+Quantile	0.1149	0.005226	0.1212	0.004716
Global+GPA	0.02122	0.001174	0.02068	0.001156
Lowess+Scale	0.1088	0.005161	0.1153	0.005067
Lowess+Quantile	0.1133	0.005085	0.1198	0.004812
Lowess+GPA	0.02215	0.001279	0.02205	0.00134

(1) **ν **and **β **: the median of variance and bias, respectively, of MSE.

**Table 2:** Comparison among different normalization methods based on simulated normal microarray data with the SIMAGE method. The data sets include 1000 genes in 50 slides with 5, 10, 30% differentially expressed genes and the ratio of up-regulated to down-regulated genes is 1:1.

**Method**	**5%**^(1)^		**10%**^(1)^		**30%**^(1)^	
	**ν**^(2)^	**β**^(2)^	**ν**	**β**	**ν**	**β**
Raw	1.677	0.06525	1.678	0.08937	1.773	0.1615
Global	0.6751	0.06319	0.4611	0.08158	0.5158	0.1697
Loess	0.2089	0.05962	0.1863	0.07056	0.1891	0.1642
Scale	1.273	0.06859	1.145	0.09515	1.368	0.1921
Quantile	0.2546	0.06579	0.2085	0.07702	0.2167	0.1825
VSN	0.2331	0.05441	0.177	0.06695	0.2014	0.1573
GPA	0.08605	0.04569	0.09839	0.06639	0.1063	0.1328
Global+Scale	0.5449	0.06481	0.3928	0.08096	0.4448	0.1851
Global+Quantile	0.08674	0.04529	0.2085	0.07702	0.2167	0.1825
Global+GPA	0.2546	0.06579	0.09967	0.06784	0.107	0.13
Loess+Scale	0.1846	0.06	0.171	0.06968	0.1788	0.161
Loess+Quantile	0.2055	0.06124	0.1852	0.07132	0.1895	0.1638
Loess+GPA	0.1324	0.0536	0.1221	0.06078	0.1291	0.1468

(1) Percentage of differentially expressed genes

(2) **ν **and **β**: the median of variance and bias, respectively, of MSE

Since the accuracy and efficiency of across-normalization method may depend on the number of replicate slides, we also conducted a series of simulation studies to verify the effect of number of replicate slides on the performance of the GPA method compared with other methods. In practice, four slides are commonly considered as the minimum number for one reliable cDNA array experiment. For this reason we have simulated microarray data from four to eight replicates. We found that, with replicate slides from 4 to 8, the GPA method yields lower MSE (sum of variance and squared bias) than other methods. In general, GPA has the best and most consistent performance in decreasing variance and bias across slides (in Global-GPA and Lowess-GPA pairs). Given these results, we recommend using the GPA method with within-slide normalization methods such as Lowess whenever feasible, especially when the number of replicate slides is smaller than four.

### Application of the GPA method for boutique arrays

Being relatively assumption-free, the GPA method may be particularly useful in analyzing boutique arrays where the majority of genes may be differentially expressed. Here we show that the GPA method is superior to the popular housekeeping gene normalization approach. We used one real apoptosis microarray data set to evaluate these two methods. The hypothesis behind this type of microarray data is that the expression of most genes changed significantly, so most normalization methods are not appropriate. Table 3 shows that our GPA normalization can yield lower replicate variability and more similar data distribution (K-S statistic) compared to housekeeping gene normalization. This is further supported in the simulated boutique arrays with two simulation methods (Tables 4, 5). The simulation data sets used here are an extreme example for boutique arrays. Approximately 60 percent of the genes are differentially expressed and three different values (5:5, 7:3 and 9:1) are applied for the ratio of up-regulated to down-regulated genes. The medians of *ν *and *β *are smaller with GPA normalization than with the housekeeping gene normalization for diverse cases. These results demonstrate that the GPA method performs better, with consistently smaller *ν *and *β *values, than the housekeeping gene method.

**Table 3:** Variance and K-S values for mouse apoptosis boutique array after GPA and housekeeping gene normalizations.

**Method**	**Mean of Variance**	**Mean of K-S Value**
Raw	0.8760382	0.700391
Housekeeping gene	0.3053415	0.620606
GPA	0.175378	0.268652

**Table 4:** Comparison between the GPA and housekeeping gene normalizations based on simulated boutique array data with the Balagurunathan's method. The data sets are simulated without or with dye bias and include 1000 genes in 10 slides with 60% differentially expressed genes. The ratios of up-regulated to down-regulated genes are 5:5, 7:3, and 9:1, respectively.

**Ratio**^(1)^	**Method**	**Without dye bias**		**With dye bias**	
		**ν**^(2)^	**β**^(2)^	**ν**	**β**
5:5	Raw	0.1147	0.008632	0.1144	0.007297
	Housekeeping gene	0.1133	0.008569	0.1164	0.007326
	GPA	0.03285	0.002833	0.03349	0.002584
7:3	Raw	0.1139	0.007859	0.1186	0.007902
	Housekeeping gene	0.1157	0.007937	0.1195	0.007427
	GPA	0.0288	0.00315	0.03133	0.003334
9:1	Raw	0.1172	0.007659	0.1178	0.007916
	Housekeeping gene	0.1187	0.006898	0.1152	0.009463
	GPA	0.02785	0.004814	0.02802	0.005119

(1) Ratio of up- to down-regulated genes

(2) **ν **and **β **: the median of variance and bias, respectively, of MSE

**Table 5:** Comparison between the GPA and housekeeping gene normalizations based on simulated boutique array data with the SIMAGE method. The data sets include 1000 genes in 50 slides with 60% differentially expressed genes and the ratios of up-regulated to down-regulated genes are 5:5, 7:3, and 9:1, respectively.

**Method**	**5:5**^(1)^		**7:3**^(1)^		**9:1**^(1)^	
	**ν**^(2)^	**β**^(2)^	**ν**	**β**	**ν**	**β**
Raw	1.467	0.4234	1.178	0.4687	1.532	0.8552
Housekeeping gene	0.2055	0.4642	0.1842	0.478	0.176	0.9346
GPA	0.111	0.3402	0.1059	0.3296	0.1194	0.5331

(1) Ratio of up-regulated to down-regulated genes

(2) **ν **and **β**: the median of variance and bias, respectively, of MSE

**Figure 4:**
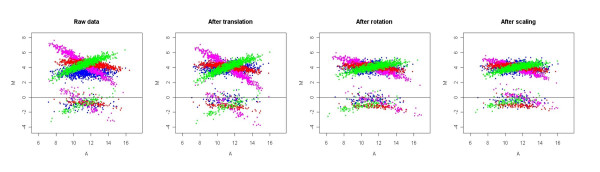
**A geometric transformation of microarray M-A plots in GPA normalization on the extreme boutique arrays**. The SIMAGE method was used to simulate the boutique array data set, which includes 50 slides with 90% up-regulated genes at 10 fold and 10% down-regulated genes at 2 fold. Four slides represented by four colours (blue, red, pink, green) were randomly selected to show their M-A plots after each GPA transformation procedure.

In order to further test the ability of GPA on boutique arrays and illustrate its advantage of being assumption-free, we simulate another extreme example of boutique arrays with 90% up-regulated genes at 10 fold and 10% down-regulated genes at 2 fold. In this case, the housekeeping gene normalization method cannot work since there are no assumed prior housekeeping genes in the experiment, whereas the GPA method can solve this problem. Figure 4 shows a geometric transformation of such extreme boutique arrays after GPA normalization procedures.

## Discussion

We demonstrate the potential of a GPA-based method for normalizing microarray data. Data normalization is an essential step for the spotted cDNA array and exerts important effects on the subsequent data analysis leading to identification of differentially expressed genes, and clustering and pathway analyses. Many normalization methods have been proposed, however, almost all the available normalization methods are based on biological or statistical assumptions about data distribution which are often not valid in practice. For example, the usual assumption that the array contains a large enough assortment of random genes, most of which are not differentially expressed across the experiments, is inherent in many existing methods including the Global, Scale, Lowess and VSN methods considered here. The assumption is particularly problematic for custom-made boutique arrays where most genes are expected to be differentially expressed, or are directly implicated in the biological process being studied. Although some novel methods have been proposed for such boutique arrays, including the housekeeping gene method [15], Zipf's law method [17] and the mixture model based method [18], these too have their own assumptions about data distribution and suffer from similar problems. Therefore normalization methods without such assumptions must be developed. We were motivated to address this question largely because we have developed a custom cDNA array [27]. With GPA normalization, there is no need to assume that expression data follow any particular distribution, for example, that the distributions of up- and down-regulated genes are symmetric.

Procrustes analysis is a powerful least-squares approach by translating, rotating and isotropic scaling to achieve a better fit among matrices of similar configurations. It has been widely applied in many fields such as statistical analysis of shapes [19], analytical chemistry [28], photogrammetry [29] and protein structural alignment [30]. Its strength in dealing with similarity among shapes or configurations led us to consider its potential for microarray data normalization analysis in balancing signal intensity level across different slides. The GPA method only requires replicate slides in the experiment, but conducting replicate experiments is a very popular experimental design since it can greatly ascertain experimental errors and reduce noise bias in the measurement and greatly help us to analyze the variability across slides.

We utilized both the real and simulated data to compare the GPA-based approach with six other normalization methods: Global, Lowess, Scale, Quantile, VSN, and one boutique array specific housekeeping gene method. For both real data sets, the GPA method showed a relatively better performance in decreasing replicate variability and increasing similarity of data distribution than other methods. Global and Scale methods performed worse in both criteria. Both VSN and Quantile performed best only in one case of variance or K-S statistic because their assumptions specifically favour one of them. Although the Lowess method can decrease variability, it showed no advantage in decreasing discrepancy of data distribution. A better performance of GPA than other across-slide based methods can still be observed on the same within-slide normalization background. The Lowess-GPA pair performed better than other dual normalizations, implying a better combination potential. Furthermore, we utilized two different parameter models to simulate several types of microarray data, which were used to accurately evaluate normalization effects through assessing true difference between known true data and normalized data. The results indicate that our GPA method outperformed other methods in reducing across-slide variability and removing systematic bias in microarray data. Overall the GPA normalization can effectively decrease the replicate variability, discrepancy of data distribution among slides and retrieve underlying biological information. It not only can be used individually to balance the bias across slides, but also in practice can be combined with other methods such as Lowess to produce better results. Moreover, the combination of GPA with Lowess can reduce discrepancies in data distribution as a result of Lowess. This is logical because Lowess and GPA have different within-slide based and across-slide based strategies. Furthermore, the application of GPA normalization in analyzing the boutique array was demonstrated in both the real and simulated boutique arrays. The GPA method not only performs consistently better than the popular boutique array-specific housekeeping gene normalization method, but can also be used where other methods are inapplicable.

## Conclusion

We have shown that GPA is a promising normalization method for microarray data analysis. In particular, GPA method is free of assumptions about data distribution inherent in other existing approaches. This makes GPA versatile and robust for diverse types of array sets, especially for custom-made boutique arrays where the majority of genes may be differentially expressed.

## Methods

### Experimental data

#### Swirl zebrafish data set

The swirl zebrafish data set is available at Bioconductor [31]. The data set has been previously utilized in several studies on normalization procedures [7,32]. The main goal of this experiment was to identify genes with altered expression in the Swirl mutant compared to the wild type zebrafish. There are 8448 genes in each slide and all experiments were replicated four times including two dye-swaps.

#### HCT116 data set

The microaray data set of HCT116 cancer cell line was retrieved from [33] which identified the dose- and time-dependent changes of gene expression in HCT116 cell lines after treatment of the topoisomerase inhibitor 1 camptothecin compound (CPT). There are 10 slides in total and each slide contains 2208 cDNA clones. This set of data was used previously in one study to compare the effectiveness of different normalization methods [34].

#### Mouse apoptosis boutique microarray

The custom boutique array data set was retrieved from [35]. The microarray experiment aimed to identify differences in apoptotic mechanisms between two different mouse cell lines. There are 5 replicated slides and each slide contains 1024 spots. The genes selected for this array are involved in apoptosis; therefore it is expected that a high proportion of genes are highly differentially expressed. The data set has been used previously for Zipf's law method in boutique microarray data normalization [17].

### Data simulation study

#### Simulation based on Balagurunathan's method

The generation of our first simulation is based on a parameterized random signal model [25], which has been utilized in several papers [24,36-41]. The precise simulation procedure is described in the Additional file 4. A series of data sets were produced with 1000 genes in 10 replicated slides. For each realization, we replicated the data set 100 times. Specific characteristics of these data sets are given below.

1. Simulation data 1 is a 1000 × 10 log ratio intensity of expression matrix. Based on the general assumption that most genes are not differentially expressed (normal array), our data was simulated to hold 3, 5, 10, and 30 percent levels of differentially expressed genes, at the same time the proportion of up-regulated genes equals that of down-regulated genes without dye bias.

2. Simulation data 2 is the same as Simulation data 1, but with dye bias which generates the banana shaped MA plot which is commonly observed in real cDNA microarray data sets.

3. Simulation data 3 is a 1000 × 10 log ratio intensity of expression matrix which is simulated as the boutique arrays in which 60 percent of genes are differentially expressed. The ratios of the up-regulated genes to the down-regulated genes are 5:5, 7:3, and 9:1.

#### Simulation based on the SIMAGE method

With the SIMAGE method [26], simulated microarray data are divided into gene expression and biases from several sources including a raw background gradient signal, a channel effect, a spot pin effect, a nonlinear effect, a quantization and saturation effect, and random error due to unknown factors. All these effects are specified in 29 parameters in SIMAGE, which were roughly estimated from real microarray data. Based on these estimated parameters, the SIMAGE method simulated microarray data mimicking the real experimental data as close as possible. The detailed input parameters in this study were described in the Additional file 5. All microarray dataset included 1000 genes in 50 replicated slides. For normal microarray data, three differential levels (5, 10, 30 %) were considered with same ratio (1:1) of up-regulated gene to down-regulated gene. For boutique array, 60 percent genes were differentially expressed and three ratios (5:5, 7:3 and 9:1) of up-regulated to down-regulated genes were considered.

### Generalized Procrustes Analysis (GPA)

The Procrustes analysis method described in this paper is available as the package vegan in the R project [42]. Scripts can be requested from the first author.

We consider the log-transformed data, log ratio intensity *M *= *log*_2_(*R*/*G*) and the mean log intensity $A=\log_{2}\sqrt{RG}$ where R and G are the red and green signal intensities respectively. We organized our log-transformed data in *N *replicated arrays as N series of matrices with *g *rows and two columns where g is the number of gene probes on the slide and the two columns are the log ratio intensity and mean log intensity on a scale (M,A). For each cDNA spot *j *in the *i*-th slide, it corresponds to a vector ${\vec{w}}_{ji}=(M_{ji},A_{ji})$ (*j *= 1,2,...,*g*; *i *= 1,2,...*N*). *M*_*ji *_and *A*_*ji *_are the measurement of M and A for gene *j *in replication *i*. Our expression level matrix of the *i*-th slide becomes the matrix $S_{i}={\left({\vec{w}}_{1i},{\vec{w}}_{2i},...,{\vec{w}}_{gi}\right)}^{T}$, where *i *= 1, 2, ..., *N*. Firstly, a reference array *S*_0 _is generated through computing the median intensity of each gene over all slides. Then each slide *S*_*i *_(*i *= 1,2,..., *N*) is translated so that its centroid is at the centroid point of the reference array *S*_0_. Let *S*_*i *_be rotated and scaled such that the residual discrepancy between *S*_*i *_and *S*_0 _is minimized. We wish to find the orthogonal matrix *H*_*i *_with (*H*_*i*_*H*_*i*_^*T *^*= I*) and scale factor *c*_*i *_so as to minimize the sum of squared distances between the corresponding points in *S*_*i *_and *S*_0_, i.e.

(1) $$\begin{array}{c} M^{2}=trace(c_{i}S_{i}H_{i}-S_{0}){\left(c_{i}S_{i}H_{i}-S_{0}\right)}^{T}\\ =c_{i}^{2}trace(S_{i}S_{i}^{T})-2c_{i}\cdot trace(L)+trace(S_{0}S_{0}^{T}) \end{array}$$

where $L=S_{i}H_{i}^{T}S_{0}^{T}$

A perfect match gives *M*^2 ^= 0. To minimize *M*^2^, we need

(2) $$c_{i}=trace(L)/trace(S_{0}S_{0}^{T})$$

*H*_*i *_= *VU*^*T*^, where *V *and *U *are products of the singular value decomposition of *S*_*i*_$S_{0}^{T}$ = *ULV*^*T *^[43].

Therefore, the output data after our Procrustes normalization method on *S*_*i *_is *S*_i_* = *c*_*i*_*S*_*i*_*H*_*i*._

In this study GPA normalization employs a reference array which is established from median values across all slides. This idea of a reference array is popularly utilized in several other normalization methods including Qspline [3,22], Iset [10], Zipf [17], and Quantile [10]. Median or mean value-based reference array is believed to be a better choice for such kind of strategy [10,44], which is also supported by our further study. Using different types of experimental and simulated data above, we found GPA normalizations with median and mean value-based reference arrays always exhibit a stable and relatively better performance than ones with individual reference arrays (details in Additional files 6, 7, 8 and 9).

The GPA method needs approximately a few seconds on a regular PC platform to normalize the data. Since the GPA algorithm computes the matrix from all the spots, it requires that all the spots have values. Here we used K-nearest neighbor averaging scheme [45] to impute missing values.

### Other normalization methods

In our study six other normalization methods were compared against our GPA-based method. These include two within-slide methods (Global [5] and Lowess [4]), three across-slide methods (Scale [4], Quantile [10] and VSN [11]) and the housekeeping gene method for boutique array [15]. All these normalization methods are available at Bioconductor [31]. To obtain the detailed algorithms and formulae used in these approaches please refer to the original references.

## Authors' contributions

HX performed all data analysis, programming and wrote the manuscript. DZ, CJM and VLT participated in the design and discussion of this study and editing the manuscript. XX proposed the original idea and supervised the project. All authors have read, and approved the final manuscript.

## Supplementary Material

Additional file 1 **The M-A plots for real swirl zebrafish data after different normalization methods**. M-A plots were used to give a general description about the effect of different normalizations on the raw microarray data. Based on different assumptions, these normalization methods result in various geometrical effects on the raw Swirl zebrafish data. Lowess method produces an ideal Lowess line along the mean of log intensity. For other methods including GPA without such Lowess type assumption, they can't make the Lowess line around zero along log ratio intensity in each plot. Global method shifts the central of log ratio intensity to zero. Scale method changes the scaling of data along the log ratio intensity directions for every slide. The M-A plots for Quantile and VSN are apparently similar to Lowess. For M-A plot after GPA normalization, the shifting and rotation of the data in each slide can be observed. Also a more obvious scaling can be observed in M-A plot of HCT116 cancer data (Figure not shown). Combinations of different methods produce combinatorial effect for plots. Click here for file

Additional file 2 The M-A plots for SIMAGE simulated microarray data after different normalization methods. This data set includes 1000 genes in 50 slides. 5 percent genes are differentially expressed. The ratio of up-regulated to down-regulated genes is 1:1. Here we showed M-A plots for first 10 slides in this data set. Click here for file

Additional file 3 The median of the two components of MSE: variance (v) and bias (*β*) in the simulated data without and with dye bias based on [25] method The levels of differential genes in data set are 3, 5, 10, 30%. The ratio of up-regulated to down-regulated genes is 1:1. Click here for file

Additional file 4 Simulation procedure of Balagurunathan's method (2002). Click here for file

Additional file 5 Input parameters used in SIMAGE simulation. Click here for file

Additional file 6 The effect of choice of a reference array on GPA normalizations. Click here for file

Additional file 7 Replicate variability and K-S statistic for the swirl zebrafish data set after GPA normalizations with different reference arrays. The upper dashed and lower straight lines indicate variability and K-S value for Lowess method, respecitively. Click here for file

Additional file 8 Replicate variability and K-S value for swirl zebrafish data after GPA normalizations based on different reference slides, and other normalization methods. Although the GPA normalizations differ based on different reference slides, the overall better performance compared to other methods can be still observed here. Click here for file

Additional file 9 The MSE results for simulated data after GPA normalizations with different reference arrays. The data is simulated by SIMAGE method, include 1000 genes in 50 slides with 5% differentially expressed genes and ratio of up-regulated to down-regulated genes is 1:1. Med represents median reference slide and Mea represents mean reference slide. Click here for file
