# Supplementary figures and images for: Using Generalized Procrustes Analysis (GPA) for normalization of cDNA microarray data
Source: BMC Bioinformatics. 2008 Jan 16;9:25. doi: 10.1186/1471-2105-9-25 (PMC2275243; doi:10.1186/1471-2105-9-25)

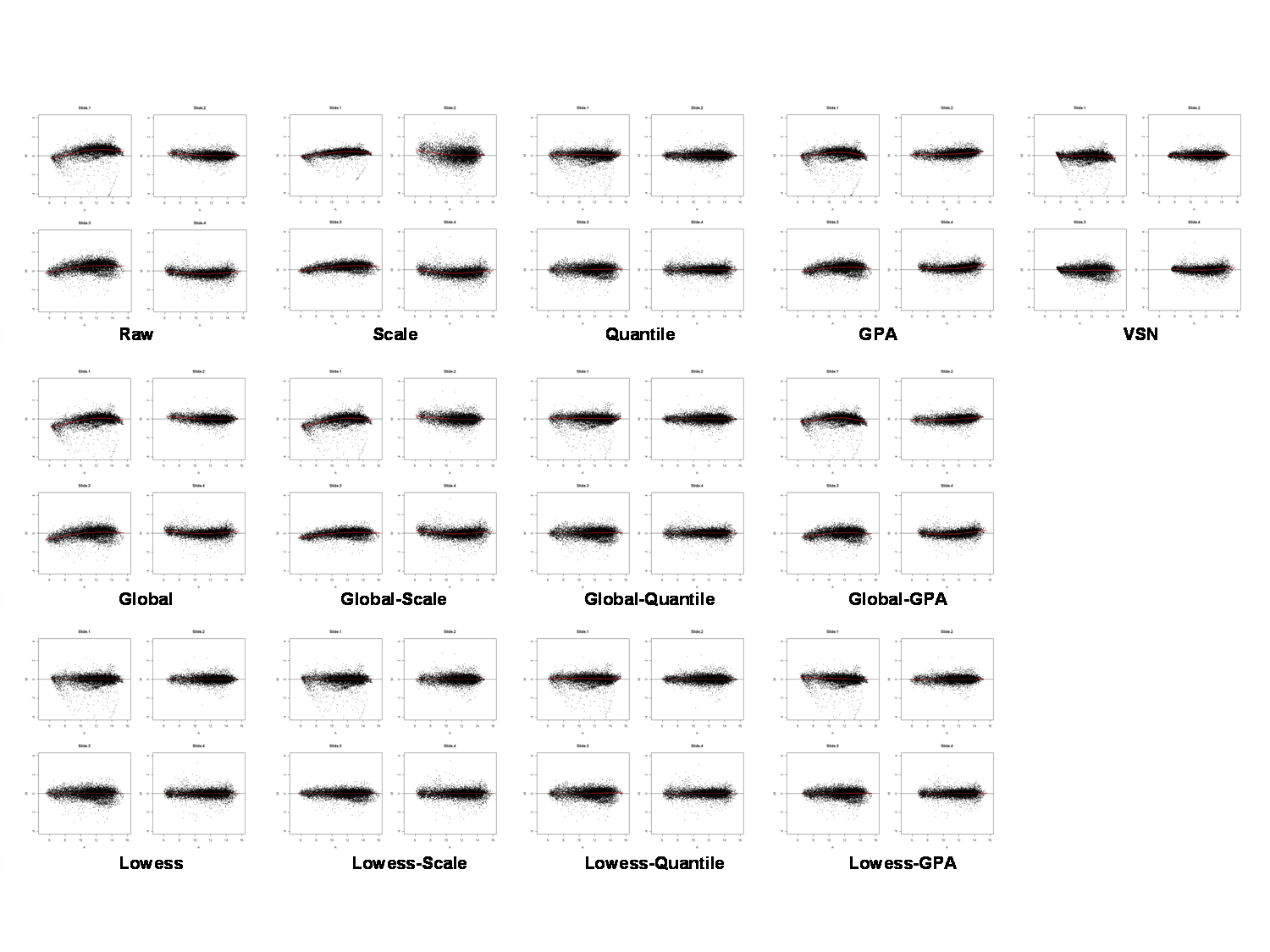

Supplement: Additional file 1 — The M-A plots for real swirl zebrafish data after different normalization methods. M-A plots were used to give a general description about the effect of different normalizations on the raw microarray data. Based on different assumptions, these normalization methods result in various geometrical effects on the raw Swirl zebrafish data. Lowess method produces an ideal Lowess line along the mean of log intensity. For other methods including GPA without such Lowess type assumption, they can't make the Lowess line around zero along log ratio intensity in each plot. Global method shifts the central of log ratio intensity to zero. Scale method changes the scaling of data along the log ratio intensity directions for every slide. The M-A plots for Quantile and VSN are apparently similar to Lowess. For M-A plot after GPA normalization, the shifting and rotation of the data in each slide can be observed. Also a more obvious scaling can be observed in M-A plot of HCT116 cancer data (Figure not shown). Combinations of different methods produce combinatorial effect for plots. [file 1471-2105-9-25-S1.TIFF]

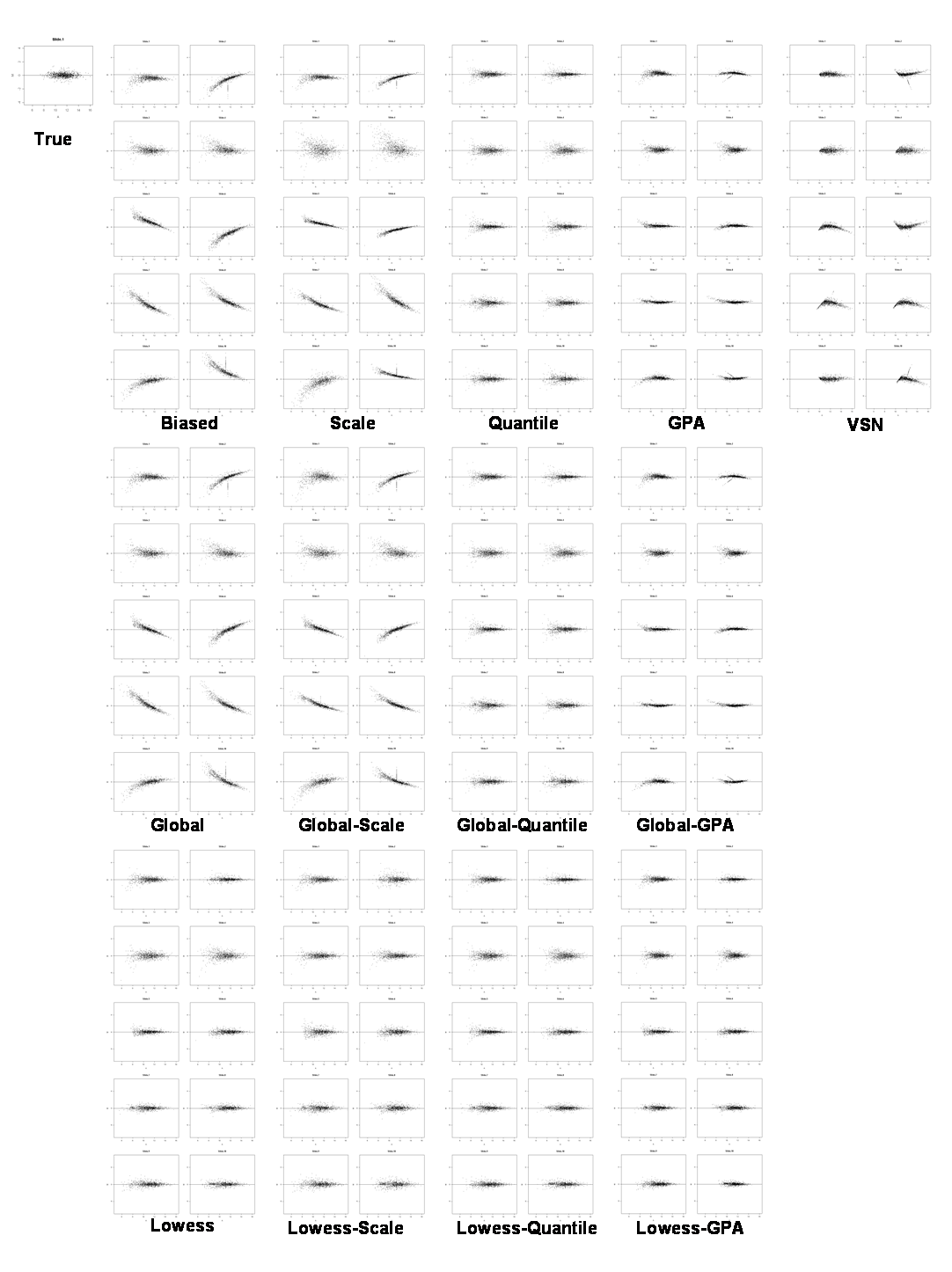

Supplement: Additional file 2 — The M-A plots for SIMAGE simulated microarray data after different normalization methods. This data set includes 1000 genes in 50 slides. 5 percent genes are differentially expressed. The ratio of up-regulated to down-regulated genes is 1:1. Here we showed M-A plots for first 10 slides in this data set. [file 1471-2105-9-25-S2.TIFF]

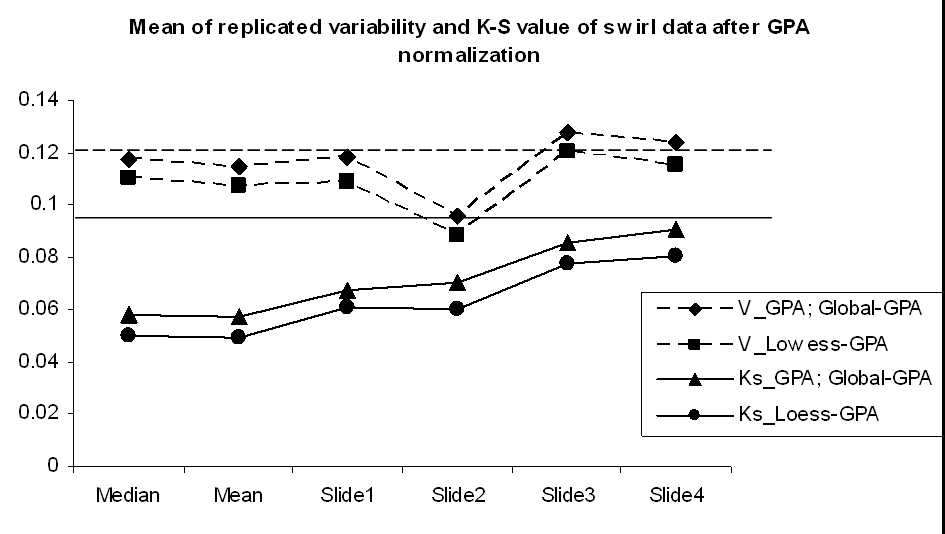

Supplement: Additional file 7 — Replicate variability and K-S statistic for the swirl zebrafish data set after GPA normalizations with different reference arrays. The upper dashed and lower straight lines indicate variability and K-S value for Lowess method, respecitively. [file 1471-2105-9-25-S7.TIFF]

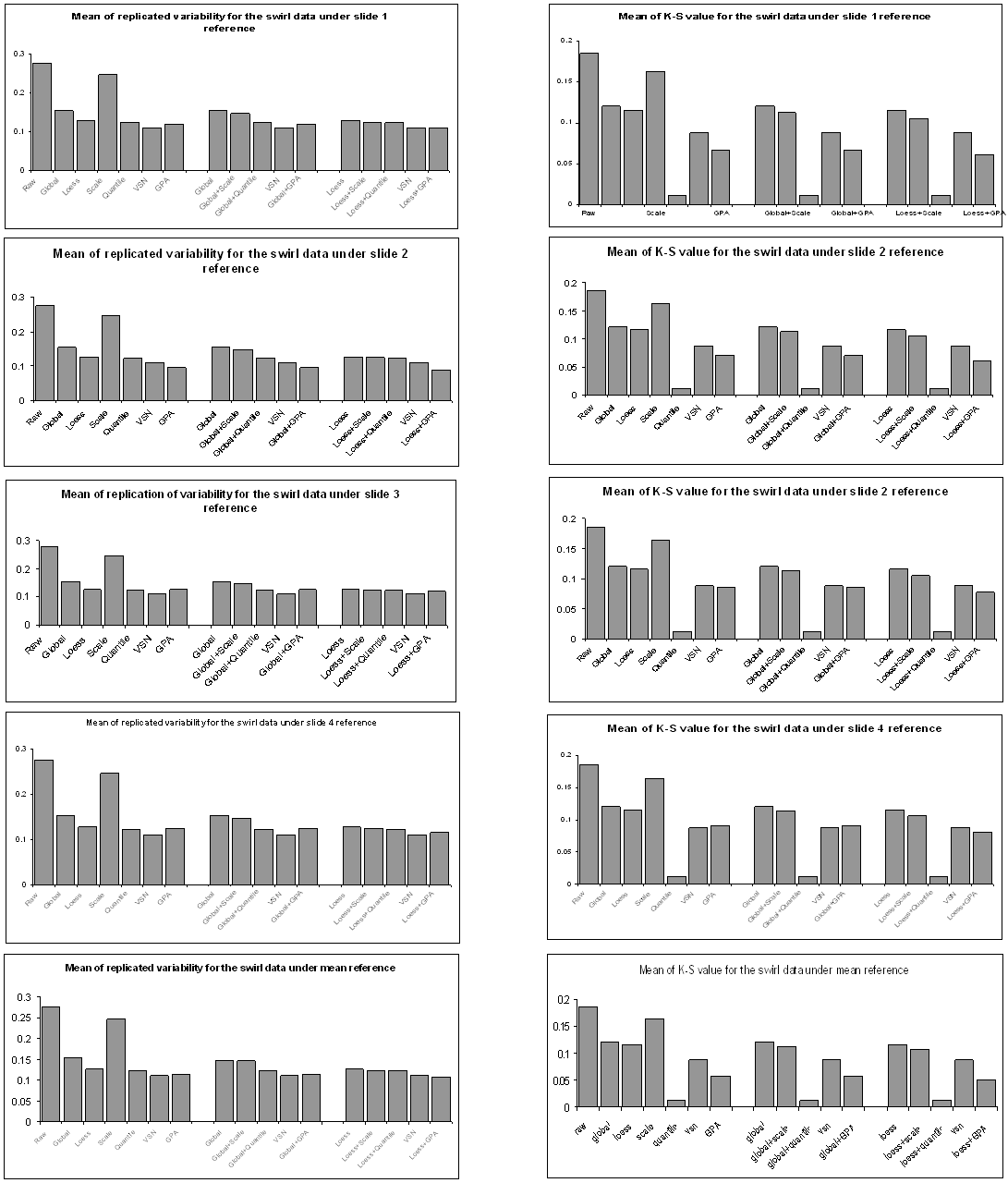

Supplement: Additional file 8 — Replicate variability and K-S value for swirl zebrafish data after GPA normalizations based on different reference slides, and other normalization methods. Although the GPA normalizations differ based on different reference slides, the overall better performance compared to other methods can be still observed here. [file 1471-2105-9-25-S8.TIFF]

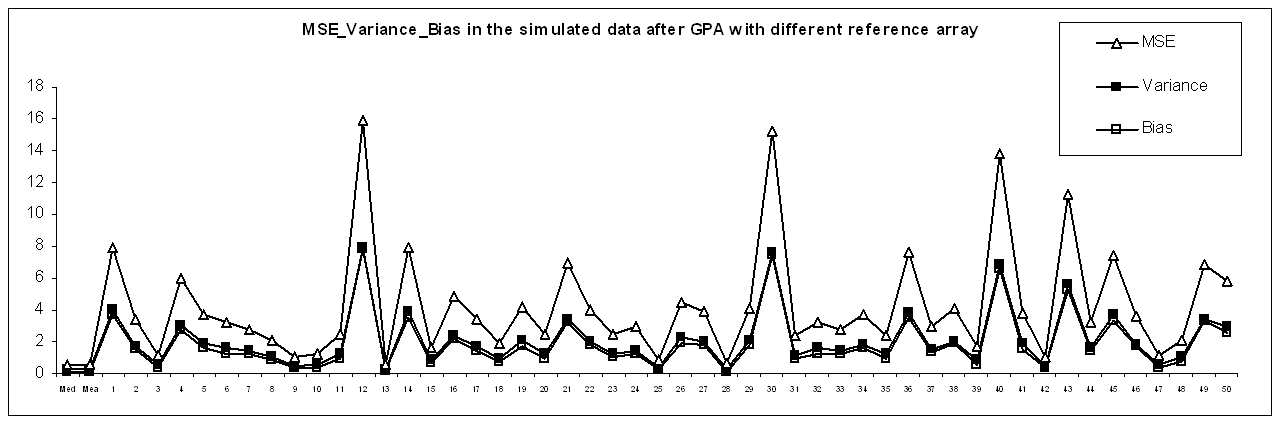

Supplement: Additional file 9 — The MSE results for simulated data after GPA normalizations with different reference arrays. The data is simulated by SIMAGE method, include 1000 genes in 50 slides with 5% differentially expressed genes and ratio of up-regulated to down-regulated genes is 1:1. Med represents median reference slide and Mea represents mean reference slide. [file 1471-2105-9-25-S9.TIFF]
