# Supplementary material for: Using Generalized Procrustes Analysis (GPA) for normalization of cDNA microarray data
Source: BMC Bioinformatics. 2008 Jan 16;9:25. doi: 10.1186/1471-2105-9-25 (PMC2275243; doi:10.1186/1471-2105-9-25)
Supplement: Additional file 3 — The median of the two components of MSE: variance (v) and bias (β) in the simulated data without and with dye bias based on [25] method The levels of differential genes in data set are 3, 5, 10, 30%. The ratio of up-regulated to down-regulated genes is 1:1. [file 1471-2105-9-25-S3.DOC]

### Additional file 3

|  |  | Without dye bias | | With dye bias | |
| --- | --- | --- | --- | --- | --- |
| **3%** | **Method** | **ν** | **β** | **ν** | **β** |
|  | None | 0.1167 | 0.004765 | 0.1256 | 0.004729 |
|  | Global | 0.1162 | 0.004637 | 0.1262 | 0.004755 |
|  | Lowess | 0.1136 | 0.004681 | 0.1195 | 0.005212 |
|  | Scale | 0.113 | 0.004671 | 0.1209 | 0.004692 |
|  | Quantile | 0.1137 | 0.004594 | 0.1199 | 0.004665 |
|  | VSN | 0.06226 | 0.002275 | 0.0607 | 0.002577 |
|  | GPA | 0.02005 | 0.001018 | 0.01958 | 0.000965 |
|  | Global +Scale | 0.1138 | 0.004752 | 0.1211 | 0.004695 |
|  | Global +Quantile | 0.1137 | 0.004594 | 0.1199 | 0.004665 |
|  | Global+GPA | 0.02004 | 0.001096 | 0.01961 | 0.000961 |
|  | Lowess+Scale | 0.1082 | 0.004747 | 0.1153 | 0.005274 |
|  | Lowess+Quantile | 0.1112 | 0.004784 | 0.1179 | 0.004802 |
|  | Lowess+GPA | 0.0204 | 0.001079 | 0.02033 | 0.001105 |
|  |  |  |  |  |  |
| **5%** | **Method** | **ν** | **β** | **ν** | **β** |
|  | None | 0.1182 | 0.004627 | 0.1231 | 0.004586 |
|  | Global | 0.1179 | 0.004732 | 0.1223 | 0.00473 |
|  | Lowess | 0.1143 | 0.005368 | 0.1182 | 0.004976 |
|  | Scale | 0.1113 | 0.004666 | 0.1201 | 0.004515 |
|  | Quantile | 0.1149 | 0.005226 | 0.1212 | 0.004716 |
|  | VSN | 0.06294 | 0.002581 | 0.06314 | 0.002541 |
|  | GPA | 0.02122 | 0.00117 | 0.02069 | 0.00119 |
|  | Global +Scale | 0.1117 | 0.004579 | 0.1191 | 0.004595 |
|  | Global +Quantile | 0.1149 | 0.005226 | 0.1212 | 0.004716 |
|  | Global+GPA | 0.02122 | 0.001174 | 0.02068 | 0.001156 |
|  | Lowess+Scale | 0.1088 | 0.005161 | 0.1153 | 0.005067 |
|  | Lowess+Quantile | 0.1133 | 0.005085 | 0.1198 | 0.004812 |
|  | Lowess+GPA | 0.02215 | 0.001279 | 0.02205 | 0.00134 |
|  |  |  |  |  |  |
| **10%** | **Method** | **ν** | **β** | **ν** | **β** |
|  | None | 0.1156 | 0.005015 | 0.1179 | 0.004732 |
|  | Global | 0.1158 | 0.004985 | 0.1181 | 0.004742 |
|  | Lowess | 0.1118 | 0.004875 | 0.113 | 0.00483 |
|  | Scale | 0.1131 | 0.005012 | 0.1134 | 0.004764 |
|  | Quantile | 0.1144 | 0.004535 | 0.1161 | 0.004574 |
|  | VSN | 0.06071 | 0.002382 | 0.06544 | 0.002405 |
|  | GPA | 0.02097 | 0.001192 | 0.02138 | 0.001229 |
|  | Global +Scale | 0.1128 | 0.004923 | 0.1142 | 0.004775 |
|  | Global +Quantile | 0.1144 | 0.004535 | 0.1161 | 0.004574 |
|  | Global+GPA | 0.02099 | 0.001145 | 0.02138 | 0.001253 |
|  | Lowess+Scale | 0.1095 | 0.004818 | 0.1102 | 0.004564 |
|  | Lowess+Quantile | 0.1126 | 0.004552 | 0.1128 | 0.004612 |
|  | Lowess+GPA | 0.02164 | 0.001222 | 0.02209 | 0.001315 |
|  |  |  |  |  |  |
| **30%** | **Method** | **ν** | **β** | **ν** | **β** |
|  | None | 0.1151 | 0.005667 | 0.1144 | 0.007297 |
|  | Global | 0.1139 | 0.005872 | 0.114 | 0.00709 |
|  | Lowess | 0.1118 | 0.006559 | 0.1123 | 0.007695 |
|  | Scale | 0.1117 | 0.006185 | 0.1144 | 0.007271 |
|  | Quantile | 0.1125 | 0.006332 | 0.1117 | 0.007321 |
|  | VSN | 0.06119 | 0.002682 | 0.0641 | 0.003004 |
|  | GPA | 0.02501 | 0.001599 | 0.03349 | 0.002584 |
|  | Global +Scale | 0.1114 | 0.00601 | 0.1148 | 0.007191 |
|  | Global +Quantile | 0.1125 | 0.006332 | 0.1117 | 0.007321 |
|  | Global+GPA | 0.02501 | 0.001594 | 0.03342 | 0.002587 |
|  | Lowess+Scale | 0.1081 | 0.006769 | 0.1094 | 0.007644 |
|  | Lowess+Quantile | 0.1113 | 0.006514 | 0.1093 | 0.007538 |
|  | Lowess+GPA | 0.02552 | 0.001653 | 0.0339 | 0.002816 |
