# Supplementary material for: Using Generalized Procrustes Analysis (GPA) for normalization of cDNA microarray data
Source: BMC Bioinformatics. 2008 Jan 16;9:25. doi: 10.1186/1471-2105-9-25 (PMC2275243; doi:10.1186/1471-2105-9-25)
Supplement: Additional file 4 — Simulation procedure of Balagurunathan's method (2002). [file 1471-2105-9-25-S4.DOC]

### Additional file 4 – Simulation procedure of Balagurunathan’s method (2002)

The generation of our simulation is based on a parameterized random signal model (Balagurunathan et al., 2002; Wang et al., 2005). More precisely, our simulation approach works as follows:

1. Simulate true expression intensity level I with exponential distribution with a mean of 3000, i.e., for *g=1*,…, the number of genes.
2. Simulate the base intensities for the red and green channels, *R_k_* and *G_k_*, respectively. Let *R_k_* ~*N*(*I_k_,α_r_I_k_*) and *G_k_* ~*N*(*I_k_,α_g_I_k_*) where *α_r_* and *α_g_* are the coefficients of variation for the intensity in the red and green channel respectively.
3. Simulate an experiment with differentially expressed genes. Randomly choose *ρ**100% genes which are outlier genes, then convert these intensities to$R_{k}^{'}=R_{k}\sqrt{t_{k}},G_{k}^{'}=G_{k}/\sqrt{t_{k}}$, for *k=1,…,* the number of the genes with $t_{k}=10^{\pm b_{k}}$where *b_k_* follow a beta distribution, *b_k_~B*(1.7,4.8) and where +/- sign means over- or under- expressed with the probability which you want. Specially, *ρ=0* when the experiment is self-self hybridized.
4. Simulate the dye-biased characteristics. Dye bias is often caused by differential labeling and detection efficiencies between the fluorescent dyes used (Uchida et al., 2005). The dyes commonly used for microarray experiments show nonlinear response characteristics, and different dyes give different responses. The fluorescent intensity is often not linearly related to the expression level. This effect is modified by the nonlinear function

$f(x)=a_{3}[a_{0}+x(1-e^{-x/a_{1}})^{a_{2}},a_{3}\geq1$ (1)

where the four parameters$a_{0},a_{1},a_{2},a_{3}$ in the formula can characterize the shape of the MA plot. Our fluorescent intensities for the two channels were transformed as $R_{k}^{"}=f(R_{k}^{'})$ and$G_{k}^{"}=f(G_{k}^{'})$ respectively.

1. Simulate background noise. The background noise for red and green channels is determined by a normal distribution, whose parameters are randomly chosen to describe the process, i.e. *I_rb_*~*N*(*I_b_,α_rb_I_b_*) and *I_gb_*~*N*(*I_b_,α_gb_I_b_*) where *α_rb_* and *α_gb_* are the coefficients of variation of the background noise for red and green channels, respectively. A signal to noise ratio is then the true mean of the signal / true mean of the background noise (*I_b_*). Our simulated intensities are corrected by subtracting the background noise.
2. Simulate the red and green foreground noise of the spots that follows with normal distribution $I_{rf}\sim N(\mu_{R_{k}^{''}},\sigma_{R_{k}^{''}}^{2})$and $I_{gf}\sim N(\mu_{G_{k}^{"}},\sigma_{G_{k}^{''}}^{2})$respectively. Then the signal intensity of each spot is

$$SR_{k}=R_{k}^{''}+I_{rf,}SG_{k}=G_{k}^{''}+I_{gf}$$

$$\sigma_{R_{k}^{"}}^{2}=a_{s_{1}}\mu_{R_{k}^{"}};a_{s_{1}}\sim U[f_{c_{1}},f_{d_{1}}]$$

$\sigma_{G_{k}^{"}}^{2}=a_{s_{2}}\mu_{G_{k}^{"}};a_{s_{2}}\sim U[f_{c_{2}},f_{d_{2}}]$ (2)

where *f_a1_, f_b1_, f_a2_, f_b2_, f_c1_, f_d1_, f_c2_, f_d2_* are given parameters.
