# Supplementary material for: Using Generalized Procrustes Analysis (GPA) for normalization of cDNA microarray data
Source: BMC Bioinformatics. 2008 Jan 16;9:25. doi: 10.1186/1471-2105-9-25 (PMC2275243; doi:10.1186/1471-2105-9-25)
Supplement: Additional file 5 — Input parameters used in SIMAGE simulation. [file 1471-2105-9-25-S5.DOC]

### Additional file 5 – Input parameters used in SIMAGE simulation

**Parameter Description Value**

Array number of grid rows 9

Array number of grid columns 4

Number of spots in a grid row 18

Number of spots in a grid column 18

Number of spot pins 12

Number of technical replicates 1

Number of genes (0 = max) 1000

Number of slides 50

Perform dye swaps no

Gene expression filter yes

Reset gene filter for each slide no

Mean signal 11.492

Change in log2ratio due to upregulation 0.832

Change in log2ratio due to downregulation 0.605

Variance of gene expression 1.775

% of differential genes (up: down=1:1) 5, 10, 30 (1:1); 60(5:5, 7:3, 9:1);

100(9:1)

Correlation between channels 0.981

Dye filter yes

Reset dye filter for each slide yes

Channel (dye) variation 0.51

Gene x Dye 0

Error filter yes

Reset error filter for each slide yes

Random noise standard deviation 0.219

Tail behaviour in the MA plot 0.09

Non-linearity filter yes

Reset non-linearity filter for each slide yes

Non-linearity parameter curvature 0.025

Non-linearity parameter tilt 0.777

Non-linearity from scanner filter yes

Reset non-linearity scanner filter for each slide yes

Scanning device bias 0.295

spotpin deviation filter yes

Reset spotpin filter for each slide no

spotpin variation 0.36

Background filter yes

Reset background filter for each slide yes

Number of background densities 5

Mean SD per background density 0.3

Maximum of the background signal (%) relative to the non-background 100

SD of the random noise for the background signals 0.1

Background gradient filter yes

Reset gradient filter for each slide yes

Maximum slope of the linear tilt 700

Missing values filter yes

Reset missing spots filter for each slide yes

Number of hairs 10

Maximum length of hair 20

Number of discs 6

Average radius disc 10

Number of missing spots 0
