# Supplementary material for: Using Generalized Procrustes Analysis (GPA) for normalization of cDNA microarray data
Source: BMC Bioinformatics. 2008 Jan 16;9:25. doi: 10.1186/1471-2105-9-25 (PMC2275243; doi:10.1186/1471-2105-9-25)
Supplement: Additional file 6 — The effect of choice of a reference array on GPA normalizations. [file 1471-2105-9-25-S6.DOC]

### Additional file 6 – The effect of choice of a reference array on GPA normalizations

The GPA normalization employs a reference array in its first step, which is also utilized in other different normalization methods including Qspline , Iset, Zipf, and Quantile. In this paper the reference slide was established from median values across all slides since several papers illustrated that median or mean value-based reference array is more robust against random variation in the data. In our study we also investigated the effect of alternative methods of choosing reference slide on the performance of GPA normalizations. Figure S1 shows the replicated variability and K-S value for swirl zebrafish data after GPA normalizations based on different reference slides. We can see that for K-S statistic, median and mean value-based reference slides provide lower values than other individual reference slides in GPA normalizations. For replicated variability, median and mean value-based reference slides showed better results than other three individual slides except slide 2. Furthermore, although the GPA normalizations differ based on different reference slides, the overall better performance compared to other methods can be still observed here (Figure S2). Similar results can also be obtained from HCT116 cancer data (data not shown) and simulated studies. For different types of simulated microarray data (5, 10, 30, 60% differential levels), GPA normalizations with median and mean value-based reference arrays always exhibit a stable and relatively better performance than ones with individual reference arrays. Figure S3 shows the MSE result for simulated data with 5% differential expressed genes after GPA normalizations. Overall, although GPA performance varied when different individual array was used as reference array, its use of median and mean value-based reference array can provide a relatively stable and better result.
